# *r*-2,*c*-6-Bis(3-methoxy­phen­yl)-*t*-3,*t*-5-dimethyl­piperidin-4-one

**DOI:** 10.1107/S1600536808023490

**Published:** 2008-07-31

**Authors:** P. Parthiban, V. Ramkumar, Nanjundan Ashok Kumar, Jong Su Kim, Yeon Tae Jeong

**Affiliations:** a Division of Image Science and Information Engineering, Pukyong National University, Busan 608 739, Republic of Korea; b Department of Chemistry, IIT Madras, Chennai, Tamilnadu, India

## Abstract

In the title compound, C_21_H_25_NO_3_, the piperidinone ring adopts a chair conformation with an equatorial orientation of all substituents; the 3-methoxy­phenyl groups make a dihedral angle of 60.26 (15)°. The carbonyl group O atom is disordered over two positions in a 0.643 (3):0.357 (3) ratio. The crystal structure is stabilized by N—H⋯O and C—H⋯O hydrogen bonding.

## Related literature

For related literature, see: Angle *et al.* (1995 ▶); Balamurugan *et al.* (2008 ▶); Gayathri *et al.* (2008 ▶); Katritzky *et al.* (1990 ▶); Ramachandran *et al.* (2007 ▶); Thiruvalluvar *et al.* (2007 ▶; Cremer & Pople (1975 ▶); Noller & Baliah (1948 ▶).
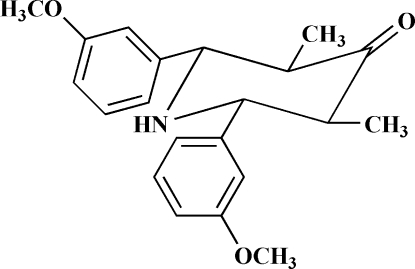


## Experimental

### 

#### Crystal data

C_21_H_25_NO_3_
*M* *_r_* = 339.42
Monoclinic, 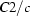

*a* = 20.9885 (6) Å
*b* = 9.7699 (2) Å
*c* = 19.8153 (5) Å
β = 109.459 (2)°
*V* = 3831.14 (17) Å^3^
*Z* = 8
Mo *K*α radiation
μ = 0.08 mm^−1^
*T* = 298 (2) K
0.25 × 0.23 × 0.22 mm

#### Data collection

Bruker APEXII CCD area-detector diffractometer
Absorption correction: multi-scan (*SADABS*; Bruker, 1999 ▶) *T*_min_ = 0.931, *T*_max_ = 0.983
23076 measured reflections
4624 independent reflections
2715 reflections with *I* > 2σ(*I*)
*R*_int_ = 0.031

#### Refinement

*R*[*F* ^2^ > 2σ(*F* ^2^)] = 0.046
*wR*(*F* ^2^) = 0.163
*S* = 0.92
4624 reflections
239 parameters
1 restraint
H atoms treated by a mixture of independent and constrained refinement
Δρ_max_ = 0.12 e Å^−3^
Δρ_min_ = −0.14 e Å^−3^

### 

Data collection: *APEX2* (Bruker–Nonius, 2004 ▶); cell refinement: *APEX2*; data reduction: *SAINT-Plus* (Bruker–Nonius, 2004 ▶); program(s) used to solve structure: *SHELXS97* (Sheldrick, 2008 ▶); program(s) used to refine structure: *SHELXL97* (Sheldrick, 2008 ▶); molecular graphics: *ORTEP-3* (Farrugia, 1997 ▶); software used to prepare material for publication: *SHELXL97*.

## Supplementary Material

Crystal structure: contains datablocks global, I. DOI: 10.1107/S1600536808023490/bx2164sup1.cif

Structure factors: contains datablocks I. DOI: 10.1107/S1600536808023490/bx2164Isup2.hkl

Additional supplementary materials:  crystallographic information; 3D view; checkCIF report

## Figures and Tables

**Table 1:** Hydrogen-bond geometry (Å, °)

*D*—H⋯*A*	*D*—H	H⋯*A*	*D*⋯*A*	*D*—H⋯*A*
N1—H1*A*⋯O1^i^	0.881 (18)	2.414 (19)	3.2784 (18)	167.1 (15)
C2—H2⋯O3^i^	0.98	2.47	3.335 (2)	146

Symmetry code: (i) 
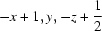
.

**Fig. 1.**
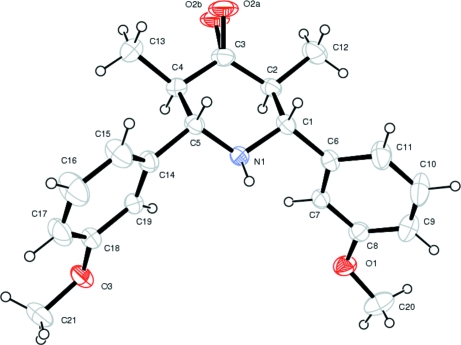
ORTEP of the molecule with atoms represented as 30% probability ellipsoids.

**Fig. 2.**
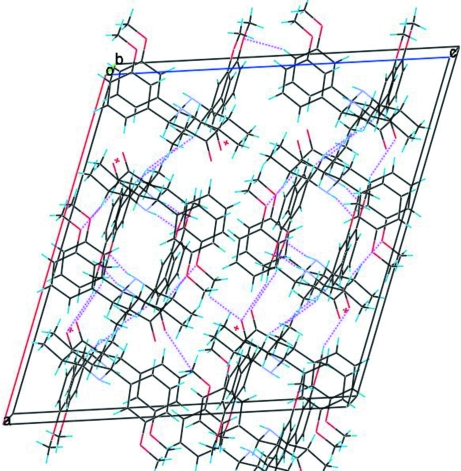
Packing of molecules along the b axis.

## supplementary crystallographic information

### Comment

Substituted piperidin-4-ones are very important class of compounds due to their presence in a wide variety of naturally occurring alkaloids, active pharmaceutical ingredients and intermediates and as building blocks of many drugs. (Angle & Breitenbucher, 1995; Katritzky & Fan, 1990). The piperidone heterocycle predominantly adopts the chair conformation (Ramachandran *et al.*, 2007; Balamurugan *et al.*, 2008) whereas depending upon the substitution, the configuration and conformation can be different (Thiruvalluvar *et al.*, 2007; Gayathri *et al.*, 2008). Hence, the present single-crystal XRD studies on the title compound have been carried out to find out the impact on the configuration and conformation of the piperidone ring due to the presence of methyl group on both sides of the carbonyl and methoxy group on the *meta* position of the phenyl rings.

In the title compound C_21_H_25_NO_3_, as shown in figure, the piperidone heterocycle adopts a chair conformation with equatorial disposition of all the substituents. The equatorial orientations of the methyl and phenyl groups are confirmed by their torsion angles. The aryl groups attached to to the piperidone ring on both sides of the secondary amino group make a dihedral angle of 60.26 (15)°.

The analysis of torsion angles, asymmetry parameters and least-squares plane calculation shows that the piperidone ring adopts chair conformation with deviation of ring atoms N1 and C3 from the C1/C2/C4/C5 plane by -0.677 and 0.563 Å;, respectively. The ring puckering parameters for N1/C1/C2/C3/C4/C5 atoms are q2 = 0.0851 (17), q3 = 0.5515, Q_T_ = 0.5580 (16) Å and θ = 8.77 (17)° (Cremer & Pople, 1975).

### Experimental

The title compound was synthesized by the one pot condensation of 2-pentanone, *meta* methoxybenzaldehyde and ammonium acetate in 1:2:1 ratio, using ethanol as a solvent by adopting the literature procedure of modified Mannich reaction, reported by Noller & Baliah (1948) for similar type compounds. The mixture was warmed and kept aside overnight. The formed 2,6-bis(3-methoxyphenyl)-3, 5-dimethylpiperidin-4-one was filtered off and washed with 1:5 ethanol, ether mixture. Thus, the obtained crude product was purified by recrystallization with ethanol to afford the colorless crystals with diffraction quality.

### Refinement

The structure was solved in the space group *C*2/*c*. The oxygen atom attached to the piperidine ring is disordered over two orientation in a 0.643 (3):0.357 (3) ratio. Nitrogen H atom was located in a difference Fourier map and refined isotropically. Other hydrogen atoms were fixed geometrically and allowed to ride on the parent carbon atoms,with aromatic C—H =0.93 Å, aliphatic C—H = 0.98Å and methyl C—H = 0.96 Å. The displacement parameters were set for phenyl and aliphatic H atoms at *U*_iso_(H) = 1.2*U*_eq_(C) and for methyl H atoms at *U*_iso_(H) = 1.5*U*_eq_(C).

### Crystal data

**Table:**

C_21_H_25_NO_3_	*F*_000_ = 1456
*M**_r_* = 339.42	*D*_x_ = 1.177 Mg m^−^^3^
Monoclinic, *C*2/*c*	Mo *K*α radiation λ = 0.71073 Å
Hall symbol: -C 2yc	Cell parameters from 5862 reflections
*a* = 20.9885 (6) Å	θ = 2.4–22.6º
*b* = 9.7699 (2) Å	µ = 0.08 mm^−^^1^
*c* = 19.8153 (5) Å	*T* = 298 (2) K
β = 109.459 (2)º	Block, colourless
*V* = 3831.14 (17) Å^3^	0.25 × 0.23 × 0.22 mm
*Z* = 8	

### Data collection

**Table:**

Bruker APEXII CCD area-detector diffractometer	4624 independent reflections
Radiation source: fine-focus sealed tube	2715 reflections with *I* > 2σ(*I*)
Monochromator: graphite	*R*_int_ = 0.031
*T* = 298(2) K	θ_max_ = 28.3º
φ and ω scans	θ_min_ = 2.1º
Absorption correction: multi-scan(SADABS; Bruker, 1999)	*h* = −27→27
*T*_min_ = 0.931, *T*_max_ = 0.983	*k* = −13→12
23076 measured reflections	*l* = −26→23

### Refinement

**Table:**

Refinement on *F*^2^	Secondary atom site location: difference Fourier map
Least-squares matrix: full	Hydrogen site location: inferred from neighbouring sites
*R*[*F*^2^ > 2σ(*F*^2^)] = 0.046	H atoms treated by a mixture of independent and constrained refinement
*wR*(*F*^2^) = 0.163	*w* = 1/[σ^2^(*F*_o_^2^) + (0.1*P*)^2^ + 0.5*P*] where *P* = (*F*_o_^2^ + 2*F*_c_^2^)/3
*S* = 0.92	(Δ/σ)_max_ < 0.001
4624 reflections	Δρ_max_ = 0.12 e Å^−^^3^
239 parameters	Δρ_min_ = −0.14 e Å^−^^3^
1 restraint	Extinction correction: none
Primary atom site location: structure-invariant direct methods	

### Special details

**Table:**

Geometry. All e.s.d.'s (except the e.s.d. in the dihedral angle between two l.s. planes)are estimated using the full covariance matrix. The cell e.s.d.'s are takeninto account individually in the estimation of e.s.d.'s in distances, anglesand torsion angles; correlations between e.s.d.'s in cell parameters are onlyused when they are defined by crystal symmetry. An approximate (isotropic)treatment of cell e.s.d.'s is used for estimating e.s.d.'s involving l.s. planes.
Refinement. Refinement of *F*^2^ against ALL reflections. The weighted *R*-factor *wR* andgoodness of fit *S* are based on *F*^2^, conventional *R*-factors *R* are basedon *F*, with *F* set to zero for negative *F*^2^. The threshold expression of*F*^2^ > σ(*F*^2^) is used only for calculating *R*-factors(gt) *etc*. and isnot relevant to the choice of reflections for refinement. *R*-factors basedon *F*^2^ are statistically about twice as large as those based on *F*, and *R*-factors based on ALL data will be even larger.

### Fractional atomic coordinates and isotropic or equivalent isotropic displacement parameters (Å^2^)

**Table:**

	*x*	*y*	*z*	*U*_iso_*/*U*_eq_	Occ. (<1)
C1	0.34428 (7)	0.63001 (15)	0.18001 (8)	0.0440 (4)	
H1	0.3033	0.6627	0.1881	0.053*	
C2	0.32387 (8)	0.52388 (16)	0.11928 (8)	0.0477 (4)	
H2	0.3651	0.4956	0.1101	0.057*	
C3	0.29531 (8)	0.39913 (18)	0.14380 (9)	0.0548 (4)	
C4	0.33555 (7)	0.33955 (15)	0.21586 (8)	0.0461 (4)	
H4	0.3776	0.3020	0.2122	0.055*	
C5	0.35427 (7)	0.45552 (15)	0.27161 (8)	0.0425 (4)	
H5	0.3127	0.4922	0.2767	0.051*	
C6	0.38064 (8)	0.75142 (15)	0.16328 (8)	0.0456 (4)	
C7	0.44600 (8)	0.73763 (15)	0.16225 (8)	0.0445 (4)	
H7	0.4668	0.6523	0.1703	0.053*	
C8	0.48077 (8)	0.84985 (17)	0.14930 (8)	0.0499 (4)	
C9	0.44996 (11)	0.97624 (18)	0.13621 (11)	0.0715 (6)	
H9	0.4730	1.0519	0.1276	0.086*	
C10	0.38482 (12)	0.9890 (2)	0.13608 (13)	0.0872 (7)	
H10	0.3637	1.0740	0.1266	0.105*	
C11	0.35003 (10)	0.87908 (19)	0.14962 (11)	0.0712 (6)	
H11	0.3060	0.8902	0.1496	0.085*	
C12	0.27530 (10)	0.5817 (2)	0.04956 (10)	0.0764 (6)	
H12A	0.2658	0.5130	0.0129	0.115*	
H12B	0.2955	0.6597	0.0353	0.115*	
H12C	0.2340	0.6089	0.0566	0.115*	
C13	0.29752 (10)	0.22367 (18)	0.23642 (11)	0.0678 (5)	
H13A	0.2563	0.2582	0.2408	0.102*	
H13B	0.3249	0.1853	0.2813	0.102*	
H13C	0.2873	0.1541	0.2001	0.102*	
C14	0.39959 (8)	0.40590 (15)	0.34391 (8)	0.0461 (4)	
C15	0.37474 (10)	0.3865 (2)	0.39968 (10)	0.0731 (6)	
H15	0.3298	0.4061	0.3934	0.088*	
C16	0.41662 (12)	0.3379 (3)	0.46462 (11)	0.0924 (8)	
H16	0.3991	0.3236	0.5014	0.111*	
C17	0.48319 (11)	0.3104 (2)	0.47607 (10)	0.0742 (6)	
H17	0.5109	0.2784	0.5203	0.089*	
C18	0.50881 (8)	0.33063 (16)	0.42127 (8)	0.0516 (4)	
C19	0.46682 (7)	0.37783 (15)	0.35542 (8)	0.0453 (4)	
H19	0.4843	0.3908	0.3185	0.054*	
C20	0.58679 (12)	0.9383 (3)	0.14770 (15)	0.0956 (7)	
H20A	0.5679	0.9847	0.1027	0.143*	
H20B	0.6315	0.9069	0.1527	0.143*	
H20C	0.5889	1.0000	0.1860	0.143*	
C21	0.62095 (10)	0.2629 (2)	0.49353 (11)	0.0866 (7)	
H21A	0.6201	0.3258	0.5305	0.130*	
H21B	0.6657	0.2595	0.4907	0.130*	
H21C	0.6082	0.1734	0.5045	0.130*	
H1A	0.4028 (8)	0.6278 (19)	0.2787 (10)	0.056 (5)*	
N1	0.38767 (6)	0.56426 (13)	0.24572 (7)	0.0424 (3)	
O1	0.54531 (6)	0.82403 (13)	0.15007 (6)	0.0624 (4)	
O2A	0.2376 (3)	0.3574 (6)	0.1104 (2)	0.0878 (14)	0.760 (15)
O2B	0.2601 (7)	0.3246 (17)	0.1015 (7)	0.0878 (14)	0.241 (15)
O3	0.57452 (6)	0.30761 (13)	0.42644 (6)	0.0666 (4)	

### Atomic displacement parameters (Å^2^)

**Table:**

	*U*^11^	*U*^22^	*U*^33^	*U*^12^	*U*^13^	*U*^23^
C1	0.0379 (8)	0.0520 (8)	0.0419 (9)	0.0083 (6)	0.0128 (7)	0.0066 (7)
C2	0.0397 (8)	0.0609 (10)	0.0385 (8)	−0.0020 (7)	0.0078 (7)	0.0035 (7)
C3	0.0445 (9)	0.0689 (11)	0.0451 (10)	−0.0112 (8)	0.0068 (8)	−0.0042 (8)
C4	0.0391 (8)	0.0486 (8)	0.0500 (9)	−0.0030 (6)	0.0141 (7)	−0.0003 (7)
C5	0.0369 (8)	0.0504 (8)	0.0408 (8)	0.0042 (6)	0.0135 (7)	0.0057 (7)
C6	0.0491 (9)	0.0479 (9)	0.0378 (8)	0.0066 (7)	0.0118 (7)	0.0070 (7)
C7	0.0465 (9)	0.0443 (8)	0.0388 (8)	0.0021 (6)	0.0090 (7)	0.0038 (6)
C8	0.0545 (10)	0.0541 (9)	0.0385 (9)	−0.0053 (7)	0.0121 (7)	−0.0006 (7)
C9	0.0908 (15)	0.0483 (10)	0.0834 (14)	−0.0055 (9)	0.0396 (12)	0.0099 (9)
C10	0.1054 (18)	0.0477 (11)	0.123 (2)	0.0209 (11)	0.0568 (16)	0.0256 (11)
C11	0.0696 (12)	0.0581 (11)	0.0941 (15)	0.0189 (9)	0.0382 (11)	0.0208 (10)
C12	0.0677 (12)	0.0969 (15)	0.0493 (11)	−0.0041 (11)	−0.0012 (10)	0.0147 (10)
C13	0.0672 (12)	0.0599 (11)	0.0748 (13)	−0.0141 (9)	0.0218 (10)	0.0068 (9)
C14	0.0486 (9)	0.0487 (8)	0.0406 (9)	0.0013 (7)	0.0145 (7)	0.0051 (7)
C15	0.0605 (11)	0.1109 (16)	0.0525 (11)	0.0134 (11)	0.0252 (10)	0.0182 (10)
C16	0.0858 (16)	0.148 (2)	0.0511 (12)	0.0181 (15)	0.0330 (12)	0.0295 (13)
C17	0.0761 (14)	0.0975 (15)	0.0408 (10)	0.0060 (11)	0.0085 (10)	0.0208 (10)
C18	0.0518 (10)	0.0504 (9)	0.0447 (10)	0.0002 (7)	0.0052 (8)	0.0068 (7)
C19	0.0481 (9)	0.0474 (8)	0.0384 (9)	−0.0018 (7)	0.0119 (7)	0.0058 (7)
C20	0.0824 (15)	0.0931 (16)	0.1168 (19)	−0.0398 (13)	0.0406 (15)	−0.0121 (14)
C21	0.0678 (13)	0.0993 (16)	0.0654 (14)	0.0113 (11)	−0.0141 (11)	0.0136 (11)
N1	0.0438 (7)	0.0441 (7)	0.0345 (7)	−0.0023 (5)	0.0066 (6)	0.0032 (6)
O1	0.0527 (7)	0.0679 (8)	0.0662 (8)	−0.0140 (5)	0.0191 (6)	0.0032 (6)
O2A	0.051 (2)	0.120 (2)	0.0707 (14)	−0.040 (2)	−0.0090 (14)	0.0095 (14)
O2B	0.051 (2)	0.120 (2)	0.0707 (14)	−0.040 (2)	−0.0090 (14)	0.0095 (14)
O3	0.0498 (7)	0.0787 (9)	0.0574 (8)	0.0069 (6)	−0.0005 (6)	0.0176 (6)

### Geometric parameters (Å, °)

**Table:**

C1—N1	1.4654 (19)	C12—H12A	0.9600
C1—C6	1.506 (2)	C12—H12B	0.9600
C1—C2	1.537 (2)	C12—H12C	0.9600
C1—H1	0.9800	C13—H13A	0.9600
C2—C3	1.508 (2)	C13—H13B	0.9600
C2—C12	1.526 (2)	C13—H13C	0.9600
C2—H2	0.9800	C14—C19	1.380 (2)
C3—O2B	1.169 (13)	C14—C15	1.383 (2)
C3—O2A	1.240 (4)	C15—C16	1.378 (3)
C3—C4	1.513 (2)	C15—H15	0.9300
C4—C13	1.517 (2)	C16—C17	1.366 (3)
C4—C5	1.539 (2)	C16—H16	0.9300
C4—H4	0.9800	C17—C18	1.377 (2)
C5—N1	1.4570 (18)	C17—H17	0.9300
C5—C14	1.511 (2)	C18—O3	1.3671 (19)
C5—H5	0.9800	C18—C19	1.388 (2)
C6—C7	1.385 (2)	C19—H19	0.9300
C6—C11	1.388 (2)	C20—O1	1.426 (2)
C7—C8	1.387 (2)	C20—H20A	0.9600
C7—H7	0.9300	C20—H20B	0.9600
C8—O1	1.3730 (19)	C20—H20C	0.9600
C8—C9	1.378 (2)	C21—O3	1.430 (2)
C9—C10	1.372 (3)	C21—H21A	0.9600
C9—H9	0.9300	C21—H21B	0.9600
C10—C11	1.374 (3)	C21—H21C	0.9600
C10—H10	0.9300	N1—H1A	0.881 (18)
C11—H11	0.9300		
			
N1—C1—C6	109.30 (12)	C2—C12—H12A	109.5
N1—C1—C2	109.18 (12)	C2—C12—H12B	109.5
C6—C1—C2	112.85 (12)	H12A—C12—H12B	109.5
N1—C1—H1	108.5	C2—C12—H12C	109.5
C6—C1—H1	108.5	H12A—C12—H12C	109.5
C2—C1—H1	108.5	H12B—C12—H12C	109.5
C3—C2—C12	111.93 (14)	C4—C13—H13A	109.5
C3—C2—C1	109.26 (12)	C4—C13—H13B	109.5
C12—C2—C1	112.75 (14)	H13A—C13—H13B	109.5
C3—C2—H2	107.6	C4—C13—H13C	109.5
C12—C2—H2	107.6	H13A—C13—H13C	109.5
C1—C2—H2	107.6	H13B—C13—H13C	109.5
O2B—C3—O2A	30.7 (7)	C19—C14—C15	118.53 (15)
O2B—C3—C2	119.8 (7)	C19—C14—C5	120.44 (13)
O2A—C3—C2	121.1 (2)	C15—C14—C5	121.03 (14)
O2B—C3—C4	117.2 (7)	C16—C15—C14	120.02 (17)
O2A—C3—C4	121.1 (2)	C16—C15—H15	120.0
C2—C3—C4	117.27 (13)	C14—C15—H15	120.0
C3—C4—C13	111.25 (13)	C17—C16—C15	121.45 (17)
C3—C4—C5	108.85 (13)	C17—C16—H16	119.3
C13—C4—C5	112.82 (13)	C15—C16—H16	119.3
C3—C4—H4	107.9	C16—C17—C18	119.18 (17)
C13—C4—H4	107.9	C16—C17—H17	120.4
C5—C4—H4	107.9	C18—C17—H17	120.4
N1—C5—C14	110.05 (12)	O3—C18—C17	124.51 (15)
N1—C5—C4	108.76 (11)	O3—C18—C19	115.71 (14)
C14—C5—C4	111.95 (12)	C17—C18—C19	119.78 (16)
N1—C5—H5	108.7	C14—C19—C18	121.04 (14)
C14—C5—H5	108.7	C14—C19—H19	119.5
C4—C5—H5	108.7	C18—C19—H19	119.5
C7—C6—C11	118.74 (15)	O1—C20—H20A	109.5
C7—C6—C1	120.24 (13)	O1—C20—H20B	109.5
C11—C6—C1	121.02 (14)	H20A—C20—H20B	109.5
C6—C7—C8	120.69 (14)	O1—C20—H20C	109.5
C6—C7—H7	119.7	H20A—C20—H20C	109.5
C8—C7—H7	119.7	H20B—C20—H20C	109.5
O1—C8—C9	124.48 (15)	O3—C21—H21A	109.5
O1—C8—C7	115.50 (14)	O3—C21—H21B	109.5
C9—C8—C7	120.02 (16)	H21A—C21—H21B	109.5
C10—C9—C8	119.12 (16)	O3—C21—H21C	109.5
C10—C9—H9	120.4	H21A—C21—H21C	109.5
C8—C9—H9	120.4	H21B—C21—H21C	109.5
C9—C10—C11	121.50 (17)	C5—N1—C1	113.77 (12)
C9—C10—H10	119.3	C5—N1—H1A	110.5 (11)
C11—C10—H10	119.3	C1—N1—H1A	108.3 (11)
C10—C11—C6	119.93 (17)	C8—O1—C20	117.83 (15)
C10—C11—H11	120.0	C18—O3—C21	118.55 (15)
C6—C11—H11	120.0		
			
N1—C1—C2—C3	51.68 (16)	O1—C8—C9—C10	179.28 (18)
C6—C1—C2—C3	173.44 (13)	C7—C8—C9—C10	−0.1 (3)
N1—C1—C2—C12	176.82 (13)	C8—C9—C10—C11	0.9 (4)
C6—C1—C2—C12	−61.42 (17)	C9—C10—C11—C6	−0.5 (4)
C12—C2—C3—O2B	32.9 (11)	C7—C6—C11—C10	−0.6 (3)
C1—C2—C3—O2B	158.5 (11)	C1—C6—C11—C10	178.52 (18)
C12—C2—C3—O2A	−2.9 (5)	N1—C5—C14—C19	−46.64 (19)
C1—C2—C3—O2A	122.7 (5)	C4—C5—C14—C19	74.44 (17)
C12—C2—C3—C4	−174.39 (15)	N1—C5—C14—C15	133.92 (17)
C1—C2—C3—C4	−48.78 (18)	C4—C5—C14—C15	−105.00 (18)
O2B—C3—C4—C13	−32.1 (10)	C19—C14—C15—C16	−1.0 (3)
O2A—C3—C4—C13	3.0 (5)	C5—C14—C15—C16	178.4 (2)
C2—C3—C4—C13	174.50 (15)	C14—C15—C16—C17	1.2 (4)
O2B—C3—C4—C5	−157.0 (10)	C15—C16—C17—C18	−0.6 (4)
O2A—C3—C4—C5	−121.9 (5)	C16—C17—C18—O3	179.6 (2)
C2—C3—C4—C5	49.58 (18)	C16—C17—C18—C19	−0.3 (3)
C3—C4—C5—N1	−53.41 (15)	C15—C14—C19—C18	0.2 (2)
C13—C4—C5—N1	−177.41 (13)	C5—C14—C19—C18	−179.21 (14)
C3—C4—C5—C14	−175.24 (12)	O3—C18—C19—C14	−179.43 (14)
C13—C4—C5—C14	60.77 (17)	C17—C18—C19—C14	0.4 (3)
N1—C1—C6—C7	50.27 (18)	C14—C5—N1—C1	−172.98 (12)
C2—C1—C6—C7	−71.42 (18)	C4—C5—N1—C1	64.05 (16)
N1—C1—C6—C11	−128.84 (17)	C6—C1—N1—C5	172.93 (12)
C2—C1—C6—C11	109.47 (18)	C2—C1—N1—C5	−63.19 (15)
C11—C6—C7—C8	1.3 (2)	C9—C8—O1—C20	9.7 (3)
C1—C6—C7—C8	−177.79 (14)	C7—C8—O1—C20	−170.90 (17)
C6—C7—C8—O1	179.56 (13)	C17—C18—O3—C21	−1.7 (3)
C6—C7—C8—C9	−1.0 (2)	C19—C18—O3—C21	178.11 (16)

### Hydrogen-bond geometry (Å, °)

**Table:**

*D*—H···*A*	*D*—H	H···*A*	*D*···*A*	*D*—H···*A*
N1—H1A···O1^i^	0.881 (18)	2.414 (19)	3.2784 (18)	167.1 (15)
C2—H2···O3^i^	0.98	2.47	3.335 (2)	146

Symmetry codes: (i) −*x*+1, *y*, −*z*+1/2.
